# A versatile injection system for flow-injection analysis

**DOI:** 10.1155/S1463924692000221

**Published:** 1992

**Authors:** M. Sollacaro, A. Dittmar, R. Later

**Affiliations:** 1 Laboratoire de Tkermorégulation et Energétique de l'Exercice CNRS URA 1341 Faculté de Médecine 8, Avenue Rockefeller Lyon Cedex 08 69373 France; 2 Laboratoire de Chimie Analytique Instrumentale Faculté de Pharmacie 8, Avenue Rockefeller Lyon Cedex 08 69373 France

## Abstract

Analyser injection systems based on the principle of flow-injection analysis depend on the technique used. They generally take the form of an injection loop valve; the injected sample volume is determined by the volume of the valve. Injection systems are seldom designed with a time factor to define this volume. The authors report on an original injection system, which enables the two techniques to be used. The paper describes the evaluation of this system using both injection techniques and the comparison between them. The results show good linearity (r = 0.999 to 1.000) and an average precision (CV = 1.04 to 1.51%) for the volume-based injection technique; (ii) good linearity (r = 1.000) and better precision (CV = 0.73 to 1.30%) for the time-based injection technique. The system can be used equally well by the loop and by the clock; however, the latter is preferable because of its practicability.


Journal of Automatic Chemistry, Vol. 14, No. 3 (May–June 1992), pp. 101–105

### A versatile injection system for flow-injection analysis

**M. Sollacaro, A. Dittmar**

*Laboratoire de Thermorégulation et Energétique de l'Exercice, CNRS URA 1341, Faculté de Médecine, 8, Avenue Rockefeller, 69373 Lyon Cedex 08, France*

**and R. Later**

*Laboratoire de Chimie Analytique Instrumentale, Faculté de Pharmacie, 8, Avenue Rockefeller, 69373 Lyon Cedex 08, France*

*Analyser injection systems based on the principle of flow-injection analysis depend on the technique used. They generally take the form of an injection loop valve; the injected sample volume is determined by the volume of the valve. Injection systems are seldom designed with a time factor to define this volume. The authors report on an original injection system, which enables the two techniques to be used. The paper describes the evaluation of this system using both injection techniques and the comparison between them. The results show good linearity (r = 0·999 to 1·000) and an average precision (CV = 1·04 to 1·51%) for the volume-based injection technique; (ii) good linearity (r = 1·000) and better precision (CV = 0·73 to 1·30%) for the time-based injection technique. The system can be used equally well by the loop and by the clock; however, the latter is preferable because of its practicability.*

### Introduction

Flow-injection analysis (FIA) is widely accepted as a means to perform rapid, reproducible and economical analyses [1,2]. The minimum equipment required is a pump, an injection system, a transport manifold and reaction tubes, a detector fitted with a low-volume flow-through cell, and a recorder. The injection system depends on the injection technique used. Ruzicka and Hansen [3] reviewed different injection techniques, the most common being 'volume-based injection' which is often achieved by an injection loop valve. The principle advantage of this technique is that the determination of the injected sample volume does not depend on the pump flow rate. Its main drawback is that it uses too much sample when the loop is filled. Relatively little information has been published about another technique: 'time-based injection'; in contrast to volume-based injection this technique allows the sample to be saved. However, in this technique, the injected volume depends on pump flow rate. Riley and co-workers [4] proposed an injection system based on such a technique: the sample was aspirated through a needle connected to a tube of the pump and the precision of the volume was defined by a pre-determined angular movement of the pump. The results showed this technique to be as efficient as those currently used.

In this paper, an original injection system is described, which can perform both kinds of injection: volume-based, timed-based. The results of the evaluation of this device for loop configuration and its comparison with the clock configuration are reported. Three characteristics: linearity, precision and carry-over effect are described.

### Instrumentation

The analyser has three parts: microcomputer, detector-recorder and a specially designed apparatus (see figure 1).

### *Microcomputer*

The analyser under microcomputer control (6502 microprocessor; Mid, Lyon, France). Communication between the microcomputer and the device is through four Versatile Interface Adapters (VIA 6522, Alpélec, Meylan, France), which process the different stages of an analysis and command each electromechanical element (electrovalves, pump, motors etc.).

In order to reduce the frequency of the microprocessor clock (1 MHz), the two timers of one VIA were used to make another clock (100 Hz). This clock, synchronized with the microprocessor clock, was used for timing the process. Programs were written in assembly language and the host program in Pascal.

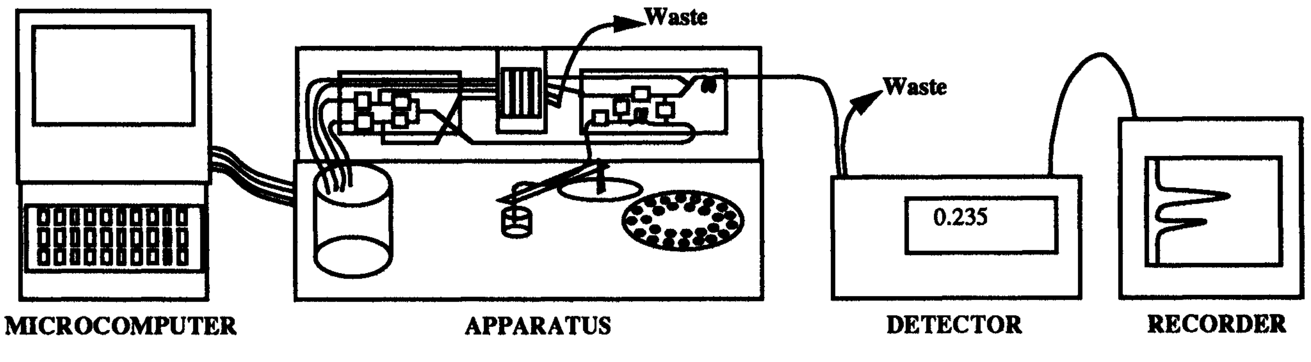

*Figure 1. The four main parts of the analyser.*

0142–0453/92 $3.00 © 1992 Taylor & Francis Ltd.

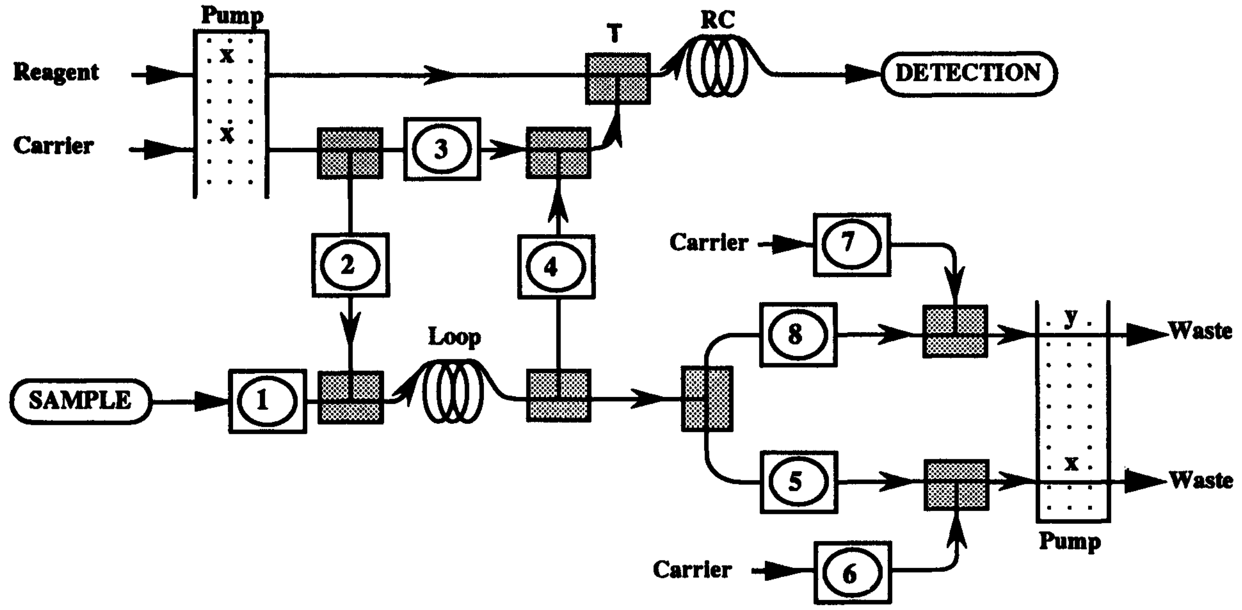

*Figure 2. Flow system (x = 1 ml/min, y = 0·17 ml/min, RC: reaction coil).*

*Detector-recorder*

The detector is a Pye Unicam SP6-500 UV spectrophotometer (Unicam, Rillieux-le-pape, France) fitted with a Hellma, QS-178, 18 µl flowcell (Hellma France, Paris, France), wavelength 340 nm. It is connected to a Linseis LS52 recorder (Linseis, Vienne, France); the sheet speed is 500 mm/h.

*Apparatus*

The apparatus has three parts: autosampler, pump-tubes and manifold.

*Autosampler*

The autosampler is made up of an arm equipped with a stainless-steel needle (0·5 mm i.d.). The arm rotates between the carrier solution cup (0·01N sulphuric acid was used in this application) and the sample vessel. The rotation is effected by a microcomputer-controlled reduction gear assembly/stepper motor (Philips R200005/HD27011, 7°30′, 12VCC; TAA, Argenteuil, France). An electromagnetized device (Mécalectro, 8.54.AA, 24 VCC; Mécalectro, Massy, France) allows two arm positions: top position for rotation, bottom position for aspiration. Sample vessels are placed on an 80-position disk. For a selected sample vessel, the disk is activated by a stepper motor (Philips HR23101, 1·8°, 5VCC) in order to reach the right position.

*Pump-tubes*

The peristaltic pump is a Gilson HP8 pump (10 rollers and eight channels; Lyon-Labo, Lyon, France). Three polyvinyl flow rate pump tubes (Bioblock Scientific, Charvieu, France) are used (inner diameters: 1·216 mm, 1·016 mm and 0·381 mm). At the given pump velocity (11·3 rounds/min), the flow rates are: 1·5 ml/min for filling the carrier solution cup; 1 ml/min for reagent stream and sample motion; and 0·17 ml/min for sampling.

*Manifold*

There are two significant features in the injection system. First, no bubble can enter into the manifold – at each arm motion, the tube connected to the needle is closed by an electrovalve 30 ms before the motion, and opened 30 ms after the needle is immersed. Second, the sample does not pass through the pump, which therefore enables better control of dispersion [5]. The following are used to construct the manifold: eight T-connectors (PT-11, Technicon; Bayer-Diagnostic, Domont, France); eight electrovalves (SIRAI, E40, 12CC; Bioblock Scientific); silicon tubes (0·5 mm i.d.); and Teflon tubes (0·5 mm i.d.) for the loops and reaction coil (100 cm).

The electrovalves permit an instantaneous control of stream movement in the flow conduits. This is achieved by squeezing and releasing silicon tubes placed under their stop-pins. The experimental procedure is controlled by the microcomputer.

### Experimental

Two methods were used for the sampling. The first was based on the use of a fixed volume Teflon tube as a loop. Changing sample volumes consists in changing physically the loop without changing the timing of the process. For the other method, the injected sample volume was determined by the sampling time and flow rate. In this case, changing sample volumes corresponds to changing the sampling time, without changing the volume of the loop and the timing of the other stages. Three sample volumes were tested for the two injection techniques: 20, 50 and 100 µl.

*Sampling using a loop circuit*

The flow system is shown in figure 2. The procedure was as follows:

(1) The electrovalves 1, 2, 4, 5 and 8 were closed while 3, 6 and 7 were opened. The arm moved in order to transfer the needle from the wash cup to the sample container.

(2) When the system was in sampling position, electrovalves 2, 4, 6 and 8 were closed while 1, 3, 5 and 7 were opened. The carrier stream passed directly to the manifold through electrovalve 3. The sample was aspirated to fill the loop and the excess drained to waste. The three Teflon loops had an i.d. of 0·5 mm and their lengths were respectively 30, 183 and 440 mm for 20, 50 and 100 µl (the connectors at each end of the loop had a total dead volume of 14 µl).

(3) In injection position, electrovalves 1, 3, 5 and 8 were closed while 2, 4, 6 and 7 were opened. The needle was transferred to the wash cup. The carrier stream passed through the loop to transport the sample to the T-connector.

(4) Electrovalves 2, 4, 6 and 8 were closed while 1, 3, 5 and 7 were opened. The needle was washed with aspirating sulphuric acid solution while the mixed reagent-sample solution passed through the reaction coil to the detector.

*Sampling using a clock*

The same manifold was used and the analytical cycle consisted of six stages:

(1) Electrovalves 1, 2, 4, 5 and 8 were closed while 3, 6 and 7 were opened. This stage corresponds to the time needed for the needle to pass from the wash cup to the sample vessel.

(2) Sampling occurred in this stage. Electrovalves 2, 4, 5 and 7 were closed while 1, 3, 6 and 8 were opened. The sample was aspirated at 0·17 ml/min flow rate through the needle during a pre-determined time given by the 100 Hz clock. The three times were, respectively, 7·06, 17·65 and 35·30 s for 20, 50 and 100 µl. The sample occupied the part of the tube located before electrovalve 1.

(3) Electrovalves 1, 2, 4, 5 and 8 were closed while 3, 6 and 7 were opened. The needle returned to the wash cup in this stage.

(4) Electrovalves 1, 3, 5 and 7 were opened while 2, 4, 6 and 8 were closed. This stage corresponded to the transport of the sample to the loop (at 1 ml/min flow rate). The length of the loop is fixed to 500 mm, i.e. 98 µl to be added to the dead volume, 14 µl. The washing solution passed through the device before and after the sample.

(5) Electrovalves 1, 3, 5 and 8 were closed while 2, 4, 6 and 7 were opened. Injection occurred in this stage. The carrier stream passed through the loop, transporting the sample to the T-connector.

(6) Electrovalves 1, 2, 4, 5 and 8 were closed while 3, 6 and 7 were opened. This last stage represented the time required for washing. The mixed reagent-sample solution passes through the reaction coil and flows to the detector.

*Reagent and samples*

0·01N sulphuric acid (SA) was used as a carrier stream and reagent, 0·5% V/V of a surfactant (BRIJ 35 Technicon; Bayer-Diagnostic) was added.

Coloured solution of potassium bichromate diluted with SA was used to prepare the different samples. For each procedure and sample volume, a coloured solution called 5Co was prepared: the concentration was experimentally adjusted to reach a measurable peak of about 0·5 OD. Four other solutions were prepared by diluting 5Co with SA. Solution 4Co: four volumes of 5Co added to one volume of SA; Solution 3Co: three volumes of 5Co added to two volumes of SA; Solution 2Co: two volumes of 5Co added to three volumes of SA; Solution 1Co: one volume of 5Co added to four volumes of SA; Solution 0Co: sulphuric acid solution.

*Validation protocols*

For each technique, three protocols were executed to measure: carry-over effect, linearity and precision.

*Carry-over study*

For the carry-over study, the following Haeckel protocol [6] was assayed: 10 series of two determinations (a1, a2) of the high concentrated solution (5Co) followed by three determinations (b1, b2, b3) of the low concentrated solution (1Co). The Wilcoxon signed rank test ($\alpha = 1\%$) was applied to the two series of b1 (polluted samples) and b3. The result of this statistical analysis determines the significance of the test on the measurement interval (0·1 OD, 0·5 OD). If this test is significant, the mean carry-over effect called h' ($h'\% = 100*(b1 - b3)/b3$) can be calculated. The two determinations a1 and a2 were used to validate the protocol on the measurement interval. This protocol was applied respecting the following conditions so that the carry-over effect h'% varies approximately from 0 to 10%. Thus, the time of the washing stage was decreased in order to increase the sampling frequency. This time was the only parameter modified by the authors and it was increased by steps of 5 s from 0 to 20 s.

The same sampling rate was chosen in order to perform the study of the linearity and the precision for the different sample volumes and injection techniques. The choice corresponds to the higher sampling rate without any carry-over effect.

*Linearity*

The five solutions (1, 2, 3, 4 and 5Co) were measured in triplicate injections. The corresponding calibration curve and the correlation coefficients were calculated (including the origin (0Co, 0 OD)), using the number of the solution (i.e.: 0, 1, 2, 3, 4 and 5) and the measurement (OD).

*Precision*

Precision was calculated through a study of repeatability which was tested by making 30 replicate determinations of solution 5Co. Thirty is the minimum sample size required to ensure normal distribution [7]. Mean, standard deviation and variation coefficient were calculated. In order to study the influence of the injected sample volume, a variance analysis is performed; in order to do this, the measurements must be normalized.

*Table 1. Volume-based injection technique.*

| Sample volume (μl) | Mean (OD) | SD (E-3 OD) | CV (%) |
|---|---|---|---|
| 20 | 0·434 | 5·98 | 1·38 |
| 50 | 0·567 | 8·57 | 1·51 |
| 100 | 0·534 | 5·53 | 1·04 |

Indeed, for any sample volume, a 5Co solution is prepared which gives a measurable peak close to an absorbance of 0·5 OD. However, these measurements are different. Therefore, the 30 values obtained for any sample volume, *Vr*, are divided by the calculated mean. Hence, the variance analysis is carried out using the three series of 30 normalized values, *Vn*.

$$Vn = \frac{Vr}{\frac{1}{30}\sum_{1}^{30} Vr}$$

### Results and discussion

### *Carry-over study*

The aim of this study was to determine the same maximum sampling rate for the two configurations, without any carry-over effect. The higher sampling rate without any significant carry-over effect was 60 samples/h for the two injection techniques. This rate was chosen in order to study the two other protocols.

### *Sampling using a loop circuit*

#### *Linearity*

For the three sample volumes, the calculated coefficients of correlation *r* range from 0·999 to 1·000. Hence, the device gives a perfect linear response between the measured solution concentration and the measurement.

#### *Repeatability*

Table 1 presents the mean, the standard deviation and the variation coefficient for the 30 determinations of solution 5Co. The variation coefficients range from 1·04 to 1·51%. In comparison with other injection systems using a spectrophotometric detection, this result corresponds to an average result. Indeed, the variation coefficient can be divided into three ranges: (1) less than 1% [8–11]; (2) between 1 and 2% [12–16], (3) more than 2% [17–19].

The lowest CV was obtained from the 100 μl sample volume. Thus, it is interesting to proceed to a variance analysis, at 99% probability level, carried out on the three injected sample volumes in order to determine whether or not one of the sample volumes presented an advantage. The result of this analysis shows no significant difference. The sample volumes presents equivalent results for the repeatability of this procedure.

*Table 2. Time-based injection technique.*

| Sample volume (μl) | Mean (OD) | SD (E-3 OD) | CV (%) |
|---|---|---|---|
| 20 | 0·511 | 6·65 | 1·30 |
| 50 | 0·543 | 6·34 | 1·18 |
| 100 | 0·493 | 6·56 | 0·73 |

This first test enables the evaluation of the injection system in a current configuration, volume-based injection technique. In light of the results, it is concluded that this injection system provides average performance.

### *Sampling using a clock*

#### *Linearity*

The calculated coefficients of correlation *r* are found to be 1·000 for the three sample volumes. Hence, as previously, the device gives a perfect linear response between the measured solution concentration and the measurement.

#### *Repeatability*

Table 2 shows the mean, the standard deviation and the variation coefficient for the 30 determinations of solution 5Co. The variation coefficients range from 0·73 and 1·30%. As previously, the lower variation coefficient was obtained by the 100 μl sample volume; a variance analysis at 99% probability level was therefore carried out on the three sample volumes. No significant difference was detected. The sample volumes present equivalent results for repeatability.

### *Comparison between the two techniques*

The two configurations gave the same result for linearity. As for repeatability, it was necessary to execute further statistical analyses in order to determine a significant difference between the two procedures. A two factor analysis of variance was used for these comparisons and no significant difference was detected. So for repeatability, these two results demonstrate that the device gives an equivalent response whatever the procedure and the injected sample volume used.

### Conclusion

The device described here gave an average performance in the case of volume-based injection, but it offerred better results for the time-based injection configuration, although no significant difference was detected. This injection system can be used equally well by a loop as by a clock. However, although volume-based injection devices are generally used, the time-based injection allows easy, fully automated analysis, and low sample volume without waste.

### References

1. Ruzicka, J. and Hansen, H. E., *Analytica Acta Chimica*, **78**, (1975), 145.

2. Pacey, G. E. and Bubnis, B. P., *Trends in Analytical Chemistry*, **6** (1987), 165.
3. Ruzicka, J. and Hansen, H. E., in *Flow Injection Analysis*, 2nd Ed., Ed. Winefordner, J. D. and Kolthoff, I. M. (Wiley, New York, 1988), p. 258.
4. Riley, C., *Clinical Chemistry*, **29** (1983), 332.
5. Ruzicka, J. and Hansen, H. E., in *Flow Injection Analysis*, 2nd Ed., Ed. Winefordner, J. D. and Kolthoff, I. M. (Wiley, New York, 1988), p. 274.
6. Haeckel, R., *Journal of Automatic Chemistry*, **10** (1988), 181.
7. Schwartz, D., (Ed), in *Méthodes statistiques à l'usage des médecins et des biologistes*, 3rd Ed. (Flammarion, Paris, 1986), p. 131.
8. Takeuchi, T., Kabasawa, Y., Horikawa, R. and Tanimura, T., *Analyst*, **113** (1988), 1673.
9. Munoz, M., Alonso, J., Bartroli, J. and Valiente, M., *Analyst*, **115** (1990), 315.
10. Nakata, R., Terashita, M., Nitta, A. and Ishikawa, K., *Analyst*, **115** (1990), 425.
11. Ferreira, J. R., Zagatoo, E. A. G., Arruda, M. A. Z. and Brienza, S. M. B., *Analyst*, **115** (1990), 779.
12. del Valle, M., Alonso, J., Bartroli, J. and Marti, I., *Analyst*, **113** (1988), 1677.
13. Masoom, M., *Analytical Letters*, **21** (1988), 2381.
14. Georgiou, C. A. and Koupparis, M. A., *Analyst*, **115** (1990), 309.
15. Szpunar-Lobinska, J., Trojanowicz, M. and Ilcheva, L., *Analyst*, **115** (1990), 319.
16. Kozuka, S., Saito, K., Oguma, K. and Kuroda, R., *Analyst*, **115** (1990), 431.
17. Laine-Cessac, P., Turcant, A. and Allain, P., *Clinical Chemistry*, **35** (1989), 77.
18. Berzas Nevado, J. J., Valiente Gonzalez, P., *Analyst*, **114** (1989), 989.
19. Pedrero Munoz, M., de Villena Rueda, F. J. M. and Polo Diez, L. M., *Analyst*, **114** (1989), 1469.

